# Autoimmume encephalitis

**DOI:** 10.1186/1546-0096-12-S1-I29

**Published:** 2014-09-17

**Authors:** Angela C Vincent

**Affiliations:** 1 Nuffield Dept of Clinical Neurosciences, University of Oxford, Oxford, OX3 9DU, UK

## 

It is now accepted that there are antibody-mediated diseases of both the peripheral and central nervous systems. Myasthenia gravis remains the prototype autoimmune disease of the neuromuscular junction, but subsequent studies have revealed antibodies to other peripheral and autonomic targets. In the 1990s, antibodies to voltage-gated potassium channel complexes were identified in acquired neuromyotonia, a condition caused by peripheral nerve hyperexcitatibility that leads to muscle fasciulations, muscle cramps and pain. Somewhat surprisingly, the same antibodies were identified in relatively acute-onset central nervous system disorders such as Morvan’s syndrome and limbic encephalitis. It turned out that the potassium channel antibodies were mainly directed at other proteins that are complexed with the channels in situ, such as LGI1 and CASPR2. These proteins help localise (CASPR2) and modify (LGI1) potassium channel function, and the antibodies bind to extracellular epitopes and are pathogenic in vitro. Tumours can be found in a proportion of each of these conditions, but the proportion varies from <10% to around 50%. Thymomas are the most common. In 2007, antibodies to NMDA receptors (NR1 principally) were identified and subsequently found quite commonly in younger patients, often women and small children, who have a very complicated form of encephalitis that results in psychiatric and movement disorders. Ovarian teratomas are common in the adult females but rare in children. Other antibodies have now been discovered, each one directed at a specific receptor or ion-channel related associated protein, although so far the associated diseases are fairly rare. Antibodies to glycine receptors are associated with a form of stiff person plus, usually termed progressive encephalomyelitis with rigidity and myoclonus (PERM), a condition which is well described in the literature and can be life threatening. Now it is recognised in more patients with a greater breadth of clinical symptoms. Each of these diseases shows a very good respond to immunotherapies such as steroids, plasma exchange, intravenous immunoglobulins. If the response is poor, second line therapies such as rituximab and/or cylclophosphamide are tried. Some require longer term immunosuppression with azathioprine or mycophenolate. Altogether there is a growing field of immunotherapy-responsive neurological diseases which need to be recognised by the clinicians and treated appropriately. There are now many neurological presentations in which the possibility of an autoimmune disease needs to be considered, and this is beginning to apply to those that are less clearly “organic”.

## Disclosure of interest

None declared.

